# Supplementary material for: Samplot: a platform for structural variant visual validation and automated filtering
Source: Genome Biol. 2021 May 25;22:161. doi: 10.1186/s13059-021-02380-5 (PMC8145817; doi:10.1186/s13059-021-02380-5)
Supplement: Supplementary file 1 — Additional file 1. Supplementary figures and supplementary text. [file 13059_2021_2380_MOESM1_ESM.docx]

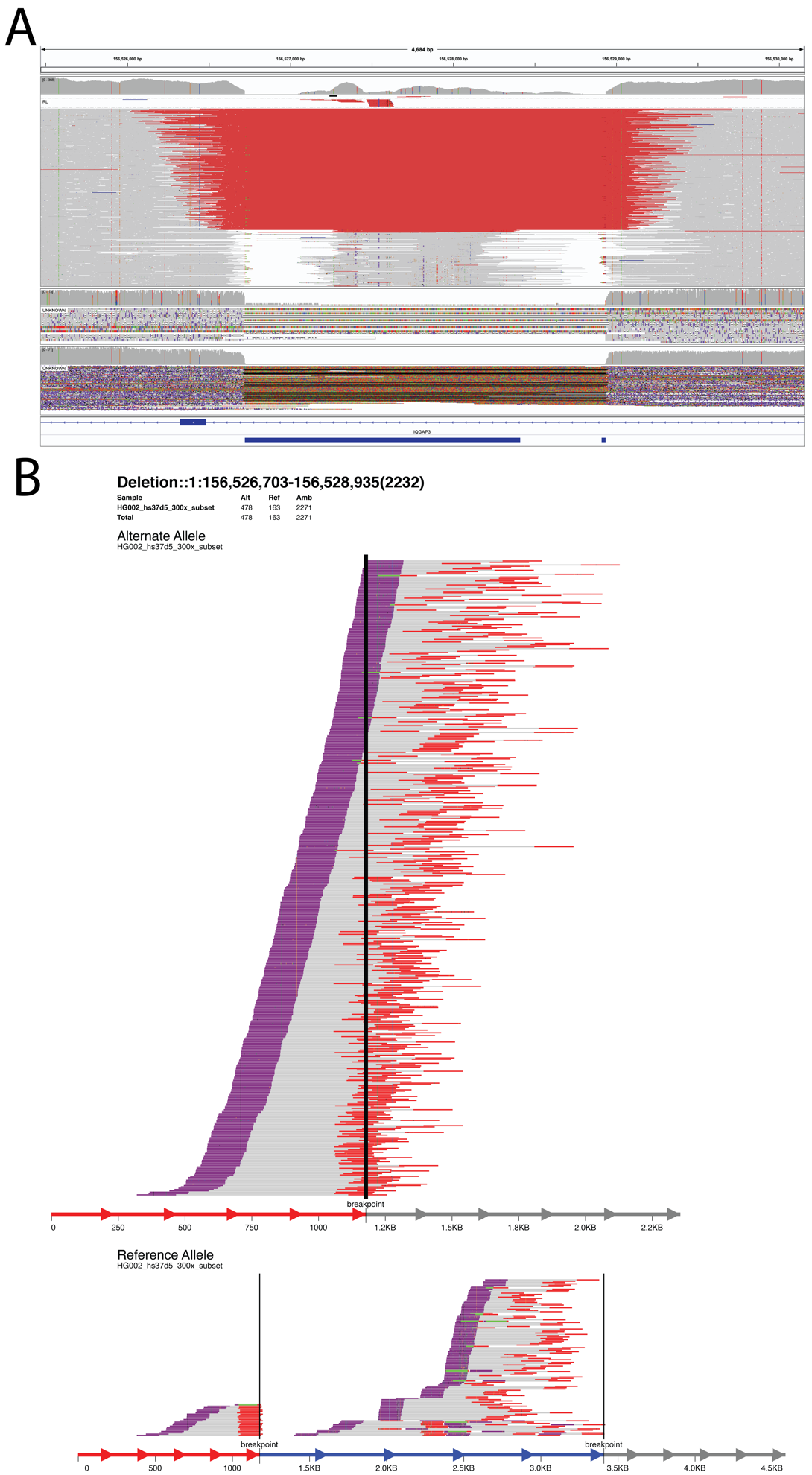


**Fig S1. Integrative Genomics Viewer and svviz deletion plots**. **A)** An IGV screenshot of the same deletion variant as shown in Figure 1. Reads are shown as pairs and sorted by insert size, with coverage shown at top of image. **B)** Svviz plot of the same deletion. Reads supporting the alternate allele are shown at top, with reads supporting the reference allele at the bottom. Breakpoints are indicated by dark vertical bars.


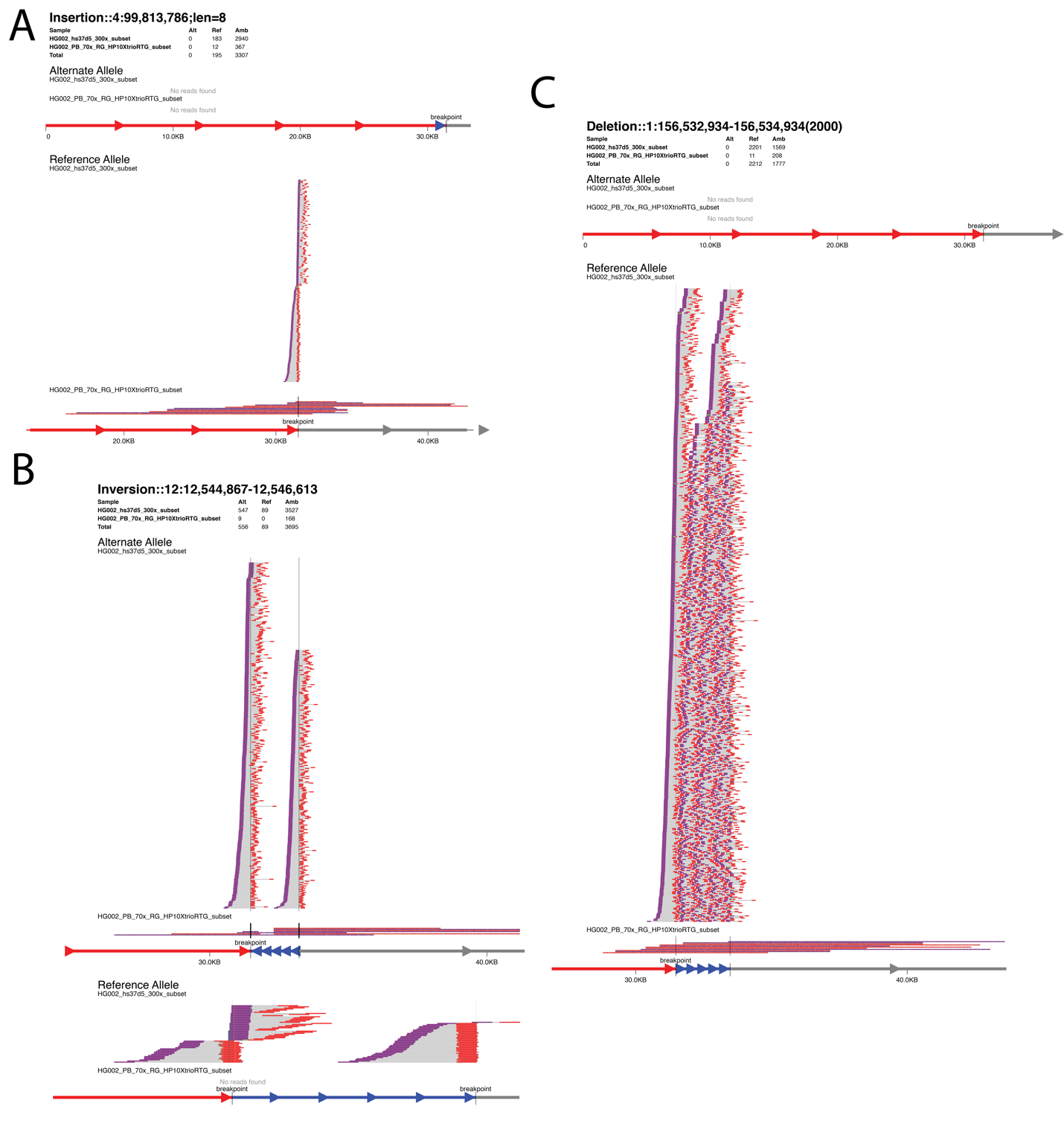


**Fig S2. Svviz images for multiple region types. A)** The duplication SV from Figure 2 plotted with svviz. B) The inversion SV from Figure 2 plotted with svviz. **C)** A region with no SV plotted with svviz.


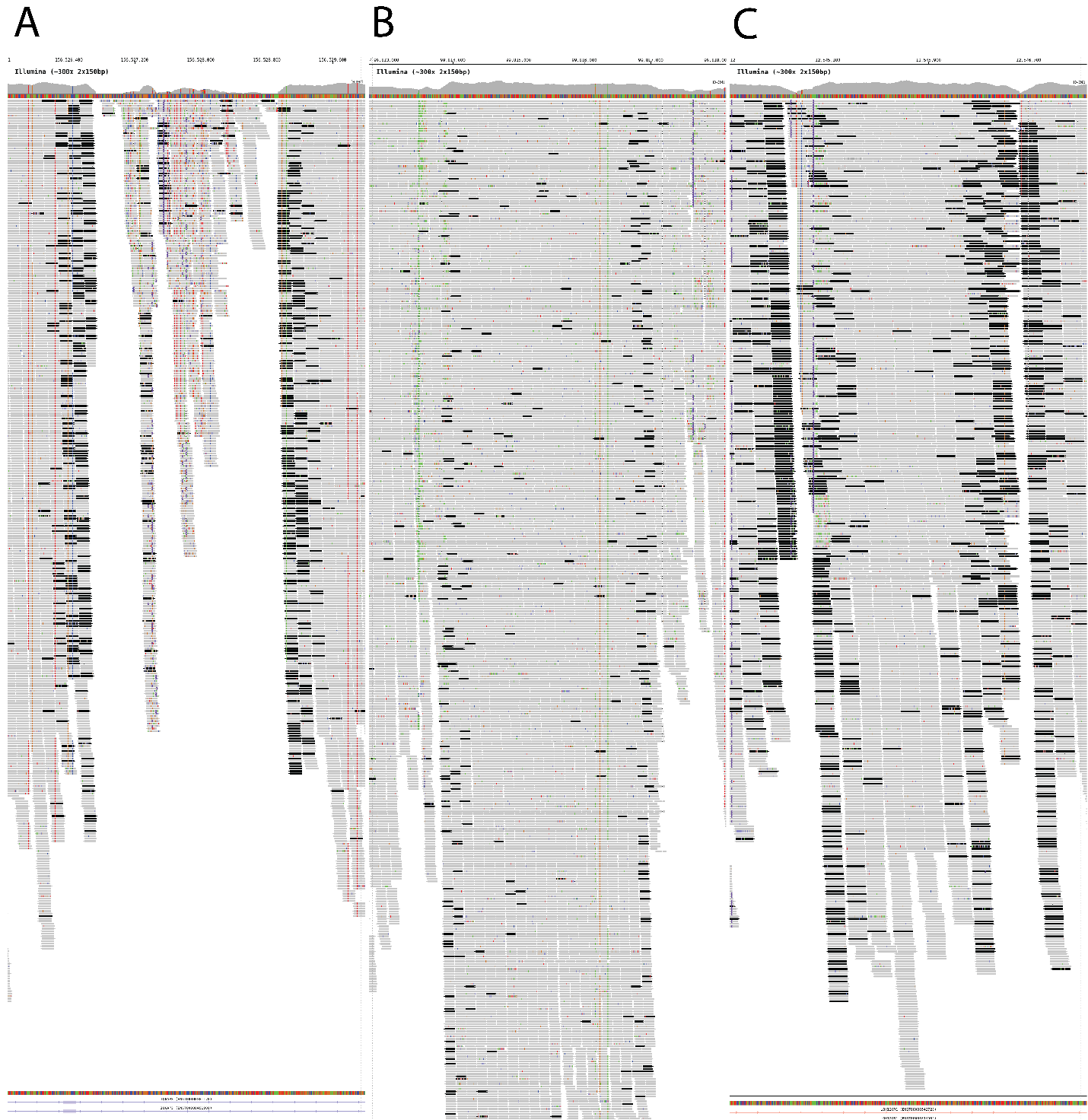


**Fig S3. Bamsnap images for multiple region types. A)** The deletion SV from Figure 1 plotted with bamsnap. B) The duplication SV from Figure 2 plotted with bamsnap (cropped to fit). **C)** The inversion SV from Figure 2 plotted with bamsnap (cropped to fit).


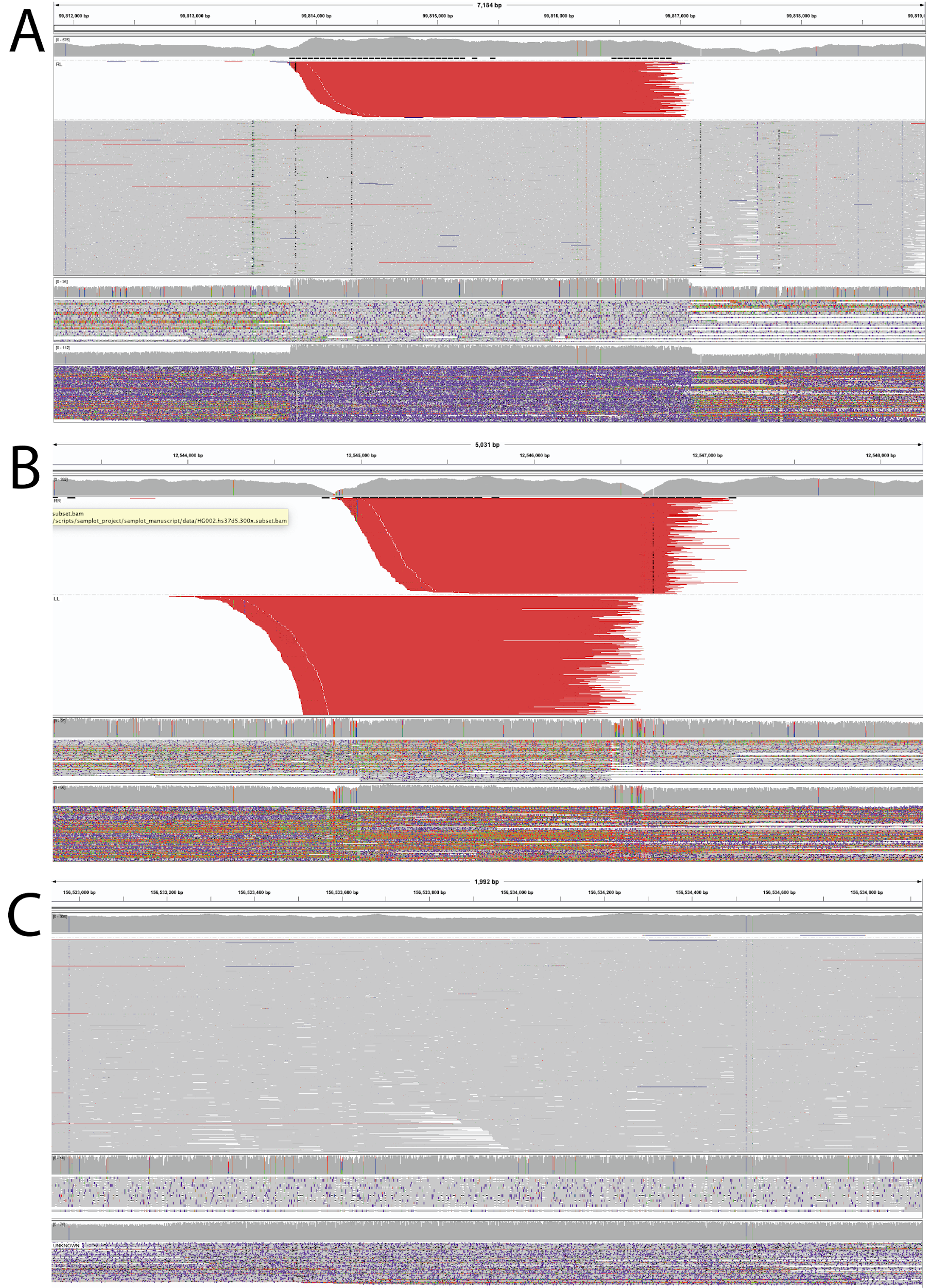


**Fig S4. IGV screenshots for multiple region types. A)** The duplication SV from Figure 2 screenshot from IGV. **B)** The inversion SV from Figure 2 screenshot from IGV. **C)** A region with no SV screenshot from IGV.

**Supplemental Figure 4. IGV screenshots for multiple region types. A)** The duplication SV from Figure 2 screenshot from IGV. **B)** The inversion SV from Figure 2 screenshot from IGV. **C)** A region with no SV screenshot from IGV.


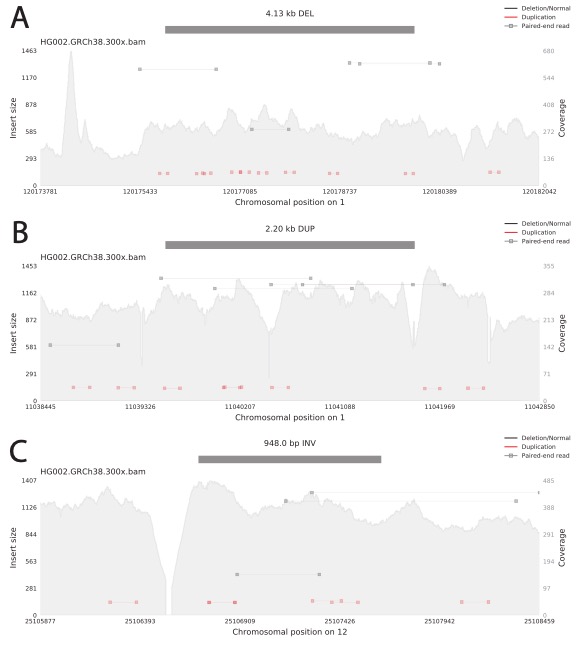


**Fig S5. A Samplot image showing false-positive SV regions. A**. A putative deletion region. **B.** A putative duplication region. **C**. A putative inversion region.


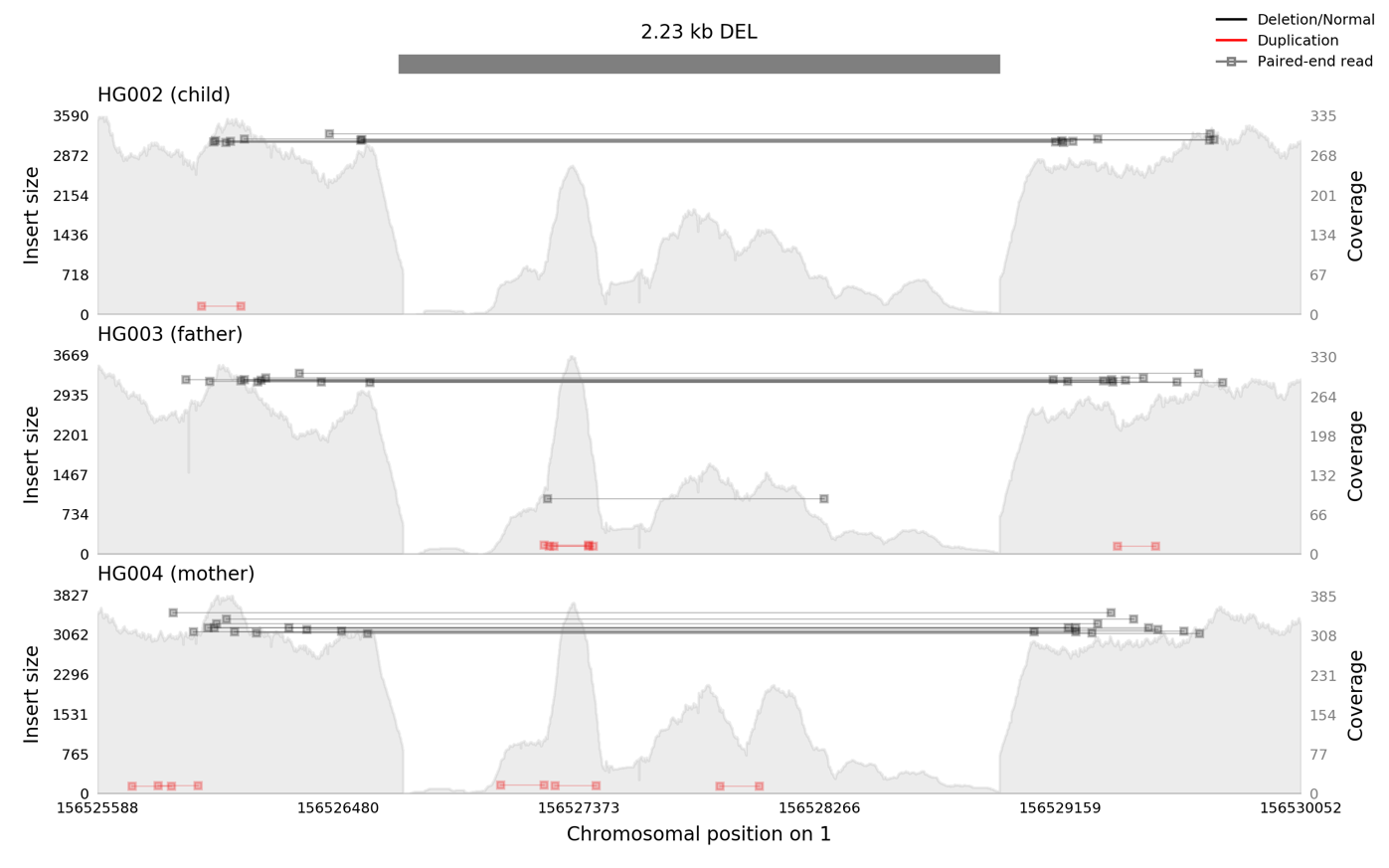


**Fig S6. A Samplot image showing a deletion variant in a trio of samples.** Evidence for the variant, in the form of loss of coverage and discordant paired-end reads, appears in the child (top) and both parents.


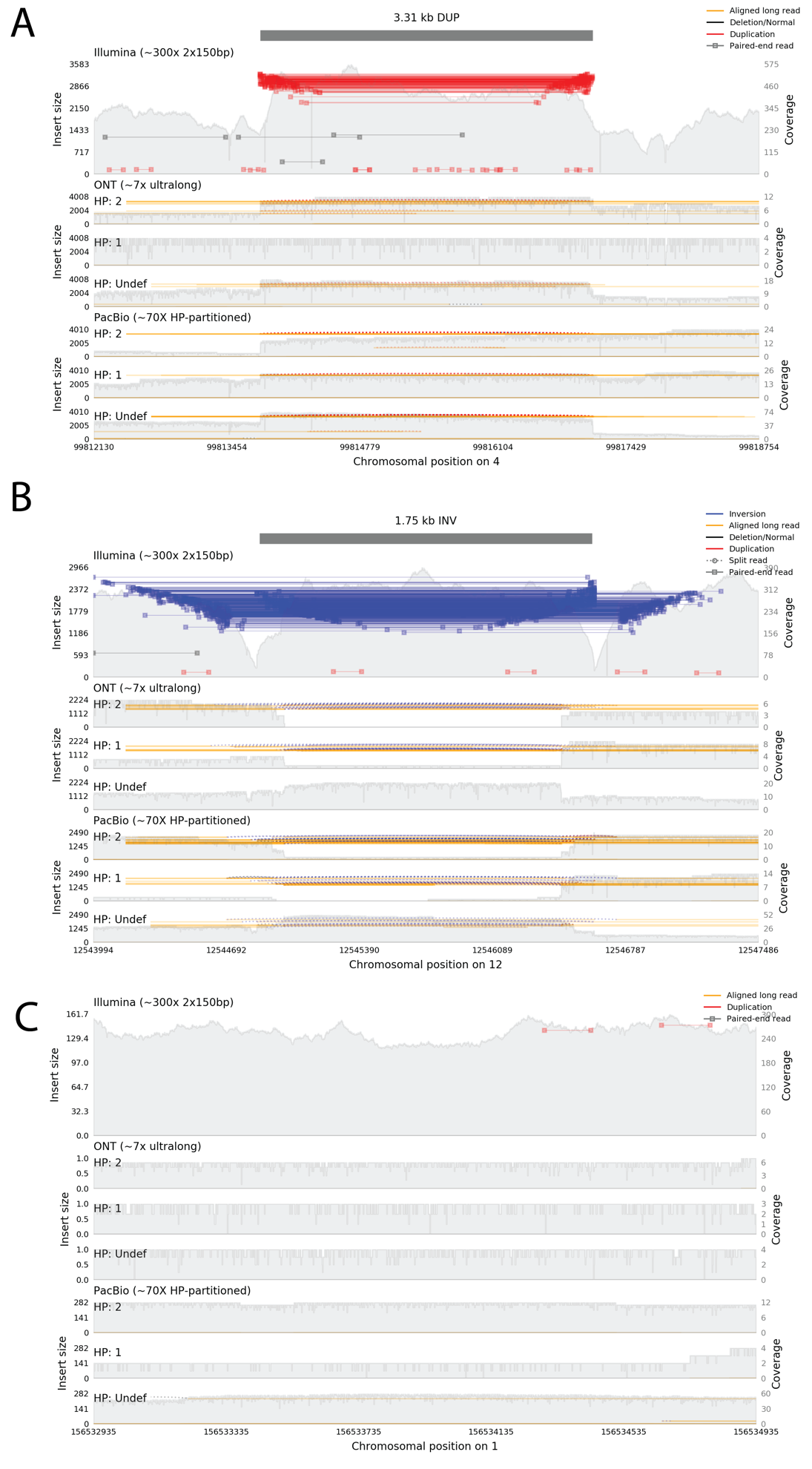


**Fig S7. Samplot images of multiple region types with multiple sequencing technologies. A)** The duplication SV from Figure 2 including Illumina, ONT, and PacBio sequence data. **B)** The inversion SV from Figure 2 including Illumina, ONT, and PacBio sequence data. **C)** A region with no SV including Illumina, ONT, and PacBio sequence data.


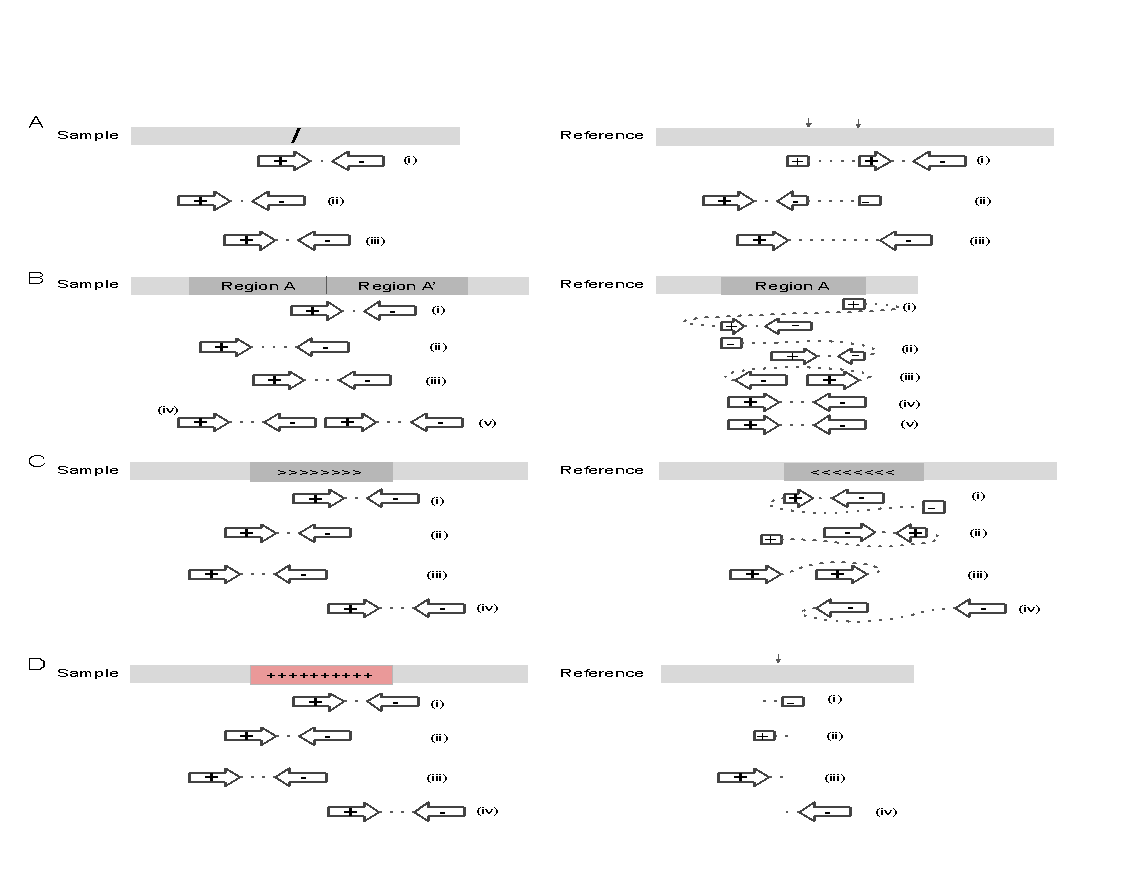


**Fig S8. Paired-end and split-read signals of structural variation.** Reads from samples with different possible SVs on the left, and the resulting reference alignments of those reads on the right. **A.** Reads from a deletion marked with “/” in the sample, coordinates marked with arrows in reference. The forward (“+”) strand end of read *i* is aligned as a split before and after the deletion. A similar split occurs in the reverse (“-”) strand end of read *ii*. Neither end of read *iii* spans the break, but the perceived insert distance when aligned to the reference genome is extended by the length of the deletion, making this a discordant read pair. **B.** Reads sequenced from a sample with a duplication of Region A also have distinct alignment patterns. Reads *i* and *ii* are aligned as splits and read iii is aligned as a discordant read pair where the read orientation is flipped and insert size increases. Reads *iv* and *v* are sequenced from duplicate copies of the same sequence, and therefore align to the same region of the reference despite coming from different locations in the sample. **C.** Reads sequenced from a sample with an inversion. Reads *i* and *ii* have split alignments with flipped orientation. Reads *iii* and *iv* are aligned as discordant pairs with the orientation of one side flipped, so read order after alignment is forward/forward (*iii*) or reverse/reverse (*iv*).


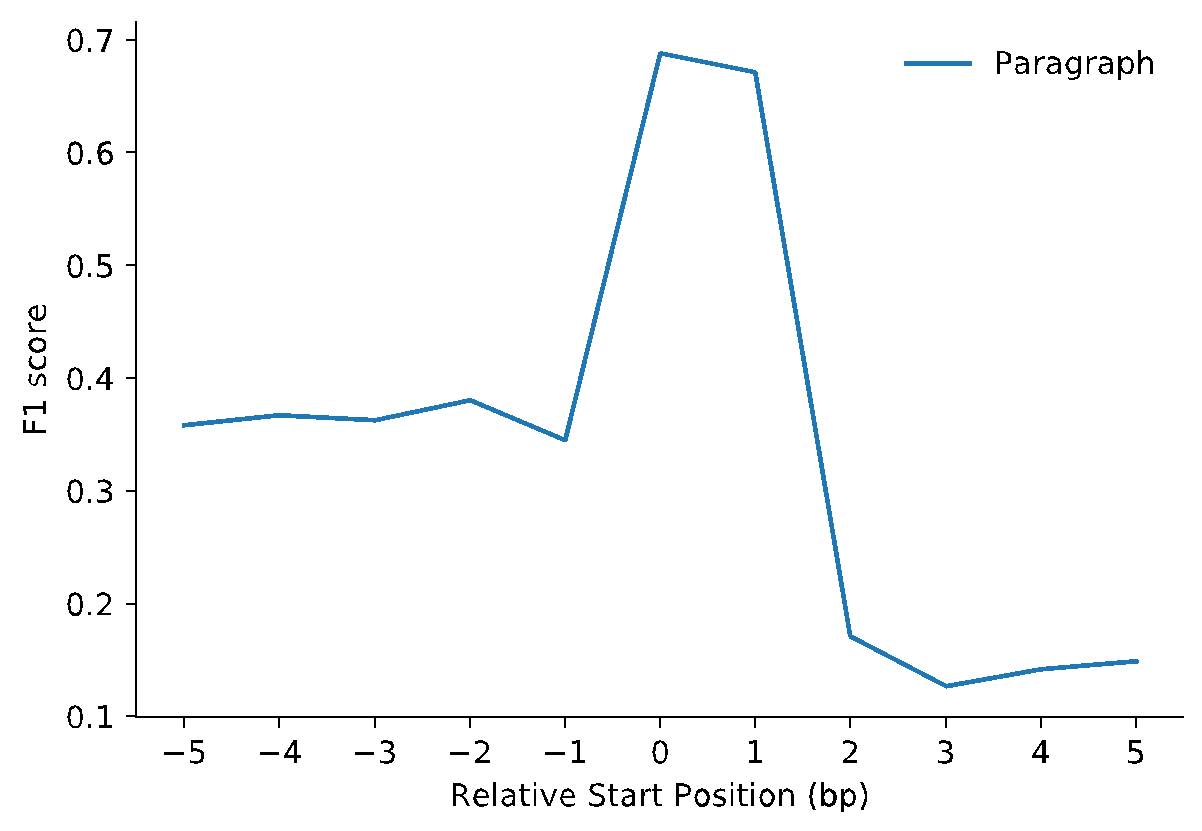


**Fig S9. Effect of breakpoint precision on Paragraph genotyping.** Paragraph’s genotyping performance, similar to other graph-based methods, is highly sensitive to breakpoint precision. Small deviations in the start position of true positive heterozygous SVs can cause Paragraph to switch genotypes from heterozygous to homozygous reference. This level of sensitivity is problematic because most of the SVs produced by short-read SV callers are not single-base resolution calls. In our experiments, MANTA’s average confidence interval size for SV start positions was 8, and LUMPY’s was 18

**Supplemental Note 1.** Samplot discordant and split alignment identification


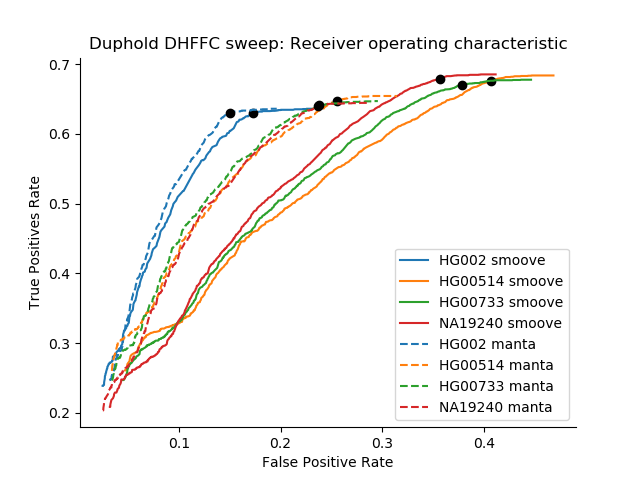


**Fig S10. DHFFC ROC curves**. Roc curves generated by varying DHFFC from 0.0 to 1.0. Black dots represent the point at which DHFFC = 0.7

**Fig S11. Reduction in False Positives vs. Reduction in True Positives. A**. DHFFC varied from 0.0 to 1.0 with the percentage reduction in True Positives and False Positives over unfiltered SV calls. **B.** Zoomed in to highlight two different DHFFC operating points. When the threshold is set to 0.7, then the reduction in false positives and true positives is 32.59% and 1.1%, respectively. If we match the reduction in positives with Samplot-ML (2.4%), then the percentage reduction in false positives increases to approximately 35.93%.


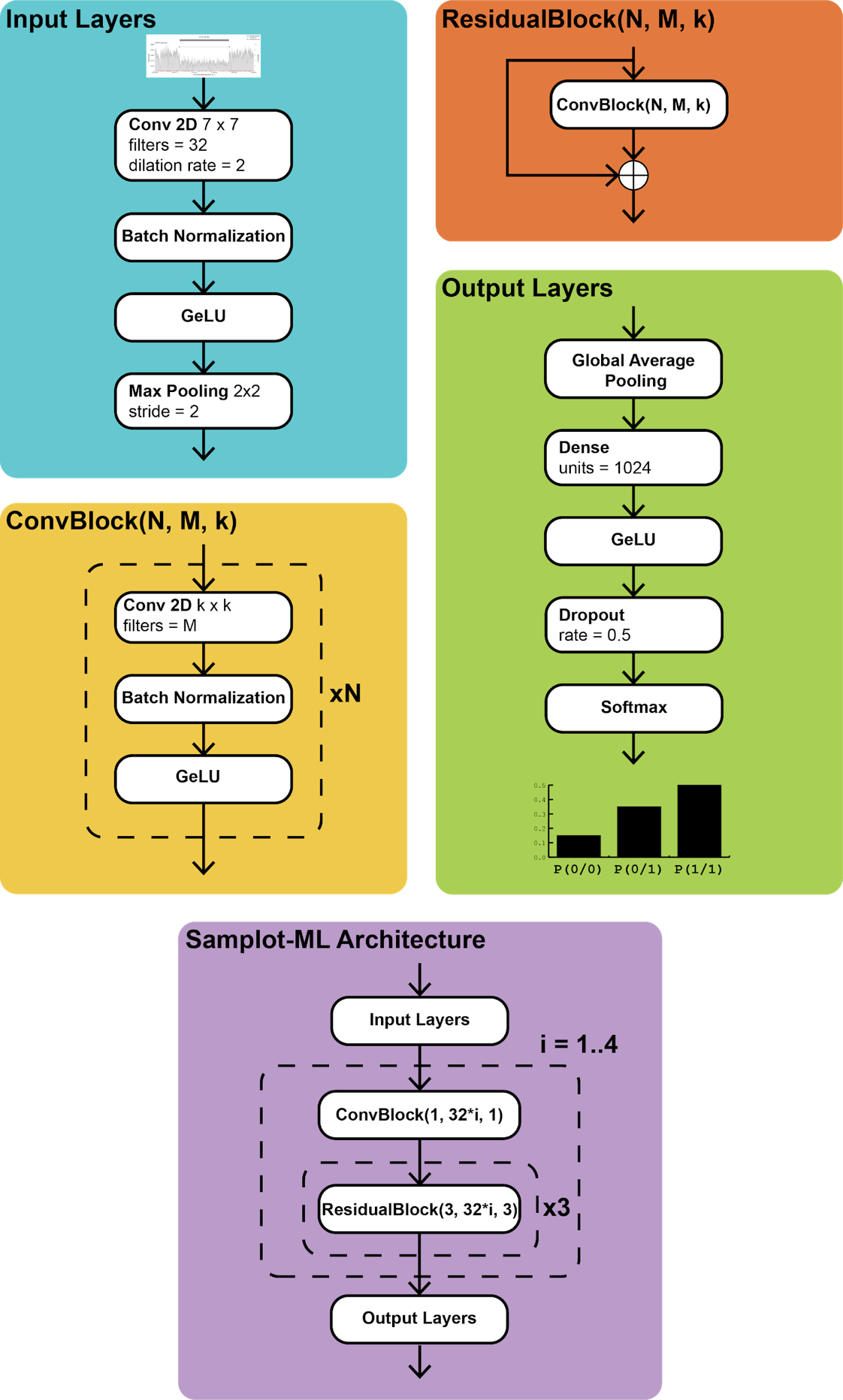


**Fig S12. Samplot-ML model architecture**. GeLU refers to the Gaussian error linear unit^34^

The Samplot SV visualization process relies on identification of read signatures which have the potential to indicate structural rearrangements. These reads are the signals used by many SV calling tools (cite lumpy, manta) to identify SVs, and in addition to deviations in depth of coverage provide the information needed by users to review putative SV calls. The primary evidence for SVs used by these tools are split or discordant read signatures. Samplot’s definitions of these are both described below:

**Split alignments** are defined in Samplot as alignments where the read contains an “SA” (Split Alignment) tag, described in the specification for BAM/CRAM files (https://github.com/samtools/hts-specs/blob/master/SAMtags.pdf). The “SA” tag indicates chimeric alignments which can be generated when part of a read aligns to one side of an SV and the rest to the other side.

**Discordant pairs** are defined in Samplot as read-pairs in which the insert size (distance between reads in a paired end read) is greater than Z standard deviations from the mean insert size, where Z is a command-line integer parameter with a default of 4. It is important to note that this definition of discordant pairs is only used to prevent the Samplot read downsampling process from removing discordant pairs, not to apply read color coding. Reads with a smaller (concordant) insert size are subject to downsampling and by default will all be removed (although still included in the depth of coverage plot). Reads with a larger (discordant) insert size are all plotted. The insert size mean and standard deviation are both calculated from the full set of reads processed within the Samplot window, which means that the size definition of a discordant read insert will scale with the size of the region of interest. The following test determines if a read pair is considered discordant:

if (read_insert_size >= mean + Z*stdev): #read is discordant

Each SV type directly supported by Samplot has a distinct read signature and is colored to match. These SV types are as follows:

**Deletions**. Split alignments from deletions have an insert distance between alignments, similar to a concordant read pair, but both alignments from a deletion-caused split read derive from the same strand (reads i and ii in panel **A**). Discordant reads that support deletions have a gap between alignments, also without changes in strand or pair order. This means that the regular pair order, in which the first member of the read pair is on the positive (+) strand and the second is on the negative (-) strand, is unaffected for discordant pairs which derive from deletions (read iii in panel **A**). Samplot does not differentiate between normal alignments and those which support a deletion, marking each with the same grey color. This allows the user to make determinations without bias.

**Duplications**. Split alignments from duplication events do not change strand, although they do change partial alignment order (reads i and ii in panel **B**). Discordant reads from duplication events cross the boundary between one copy of the duplicated region and the next. This means that the pair order is swapped, resulting in the first in pair being a negative strand read, while the second in pair is a positive strand read (read iii in panel **B**). Reads from entirely within the duplication are indistinguishable from those from the initial copy (reads iv and v in panel B). Reads identified as potentially supporting a duplication are colored red in Samplot. Note: Samplot defines duplications as tandem duplications, while other types of duplication event are considered insertions and are not directly supported.

**Inversions.** Split alignments from inversions undergo a switch in alignment direction for the partial alignment that is read from the inverted region (reads i and ii in panel **C**). Discordant reads from inversions similarly change alignment direction, resulting in both reads from a pair being aligned to the same strand (+/+ or -/-, reads iii and iv in panel **C**). These reads are colored blue. One distinguishing factor in inversion signals is that each breakpoint of the inversion presents a separate collection of reads, with +/+ orientation or -/- orientation. This is often visible in Samplot inversion plots.

**Translocations.** Interchromosomal events can take any of the above forms and thus may be colored grey, red, or blue as indicated by alignments.

Although each of these descriptions is based on the operations used to define and color short, paired-end reads, the long read processing is very similar. Each gapped alignment in a long read is considered a potential structural event, with a minimum event size defined by default as 20 base pairs. This can be modified by the user via command-line parameter. Each alignment is placed on the vertical axis of the Samplot image based on the longest alignment event within the genomic window plotted and the event is colored following the same rules above, with deletions in grey, duplications in red, and inversions in blue. The rest of the long read is colored orange, differentiating long from short reads.

### **Supplemental Note 2.** Running Samplot-ML

From the Samplot-ML root directory:

**1. Generate Images from VCF of deletion SVs**

bcftools query -f '%CHROM\t%POS\t%INFO\END\n' **$vcf_path** |

gargs -p **$n_processes** \

"bash data_processing/gen_img.sh \\

--chrom {0} --start {1} --end {2} \\

--sample $sample --genotype DEL \\

--min-mqual 10 \\

--fasta **$fasta_reference** \\

--bam-file **$bam** \\

--out-dir **$out**

gargs is an open source alternative to xargs and can be found at <https://github.com/brentp/gargs>

**2. Crop Images**

bash data_processing/crop.sh \

-p **$n_processes** \

-d **$path_to_images \**

-o **$out**

**3. Filter input VCF/modify predicted genotypes with Samplot-ML**

find **$path_to_cropped_images** -name '*.png' > image-list.txt

bash evaluation/create_test_vcfs.sh \

--model-path saved_models/samplot-ml.h5 \

--data-list image-list.txt \

--vcf **$vcf_path** \

--num-processes **$n_processes** \

--batch-size **$batch_size** \

--out-dir **$out**

**$n_processes** is the number of cpu processes used to load images to the model. **$batch_size** is the number of images to feed to the model at once. The output directory will contain the filtered vcf and a bed file with regions and prediction scores with format:

Chrom start end Pref Phet Palt

Where Pref, Phet, and Palt are the prediction scores for homozygous reference, heterozygous, and homozygous alternate genotypes.
