# Supplementary material for: Samplot: a platform for structural variant visual validation and automated filtering
Source: Genome Biol. 2021 May 25;22:161. doi: 10.1186/s13059-021-02380-5 (PMC8145817; doi:10.1186/s13059-021-02380-5)
Supplement: Supplementary file 7 — Additional file 7. Review history. [file 13059_2021_2380_MOESM7_ESM.docx]

Review History

**First round of review**

**Reviewer 1**

**Comments to author:**

Review of Belyeu. et al.

The authors present a method, SamPlot, to render and validate SVs either manually or with machine learning. I have been using SamPlot in my research and confirm the manual curation is easy to install and use, and is useful. Normally we would use IGV in a batch mode, but it is painfully slow, and the renderings of samplot are easier to view. Most of my comments are minor, with the one major comment regarding the comparison with duphold.

Comment 1. (minor) After stating SamPlot-ML only supports deletions, it is stated that other SV classes will be supported in the future. Because this is speculation, it should be moved to the discussion. You can use the active form to introduce SamPlot-ML: We developed SamPlot-ML to classify deletion SV calls …

Other statements in the manuscript are forward looking, and should not be used unless in the discussion, for example, "We are exploring other training strategies, including using regions that appear to be mutated from short-read data but are normal in long-read data." in the methods.

Comment 2 (minor) The figure 4A would be more interpretable as confusion matrices -- what are the SamPlot-ML classifications relative to GIAB?

Comment 3 (minor) Typo - CHM1/CHM3 are stated to be "diploid". You'll want to say they are homozygous diploid, or 46,XX. Most of the time it is referred to as a haploid cell line which is technically incorrect.

Comment 4 (minor) Conclusions should be folded into discussion.

Comment 5 (major). While the performance of paragraph and SV2 are surprisingly abysmal and sufficiently presented, the comparison with duphold is somewhat broad. For example, if parameters of duphold could be changed to reduce true positives from 1.1% to 2.4%, how many false positives are removed? Are there any particular regions where the ML approach correctly classifies a call when duphold does not? Additional effort should be made to highlight these regions (overlapping mobile elements/ tandem repeats / segmental duplications , genes, etc.).

Comment 6 (minor/major). The long read data is presented as "proof of concept". To me, this does not mean ready for publication, and due to the importance of having a tool to validate/curate calls from large sequencing projects such as TOPMed, the paper stands on it's own with short read data. It is up to the editors to decide if proof of concept results are appropriate to publish.

**Reviewer 2**

**Comments to author:**

The authors describe their software, samplot, for visualising alignment-and read depth-based evidence of structural variants. Samplot supports manual visual curation of SV calls, which can be employed to reduce false positives in some situations. Motivated by the idea of visually inspecting supporting evidence, the authors have also developed a machine learning model, samplot-ml, to automate the removal of false positive calls. This uses a convolutional neural network to perform classification on the images produced by samplot. Samplot-ml currently supports deletions only. A proof-of-concept model was also developed for long reads.

In my view, samplot is a useful tool for when the evidence for an SV need to be inspected.

This is a task that arises (i) in the wide-scale manual curation of SVs from an individual sample—an approach that is sometimes applied; (ii) in targeted ways to look at potential causal variants in trio studies; (iii) in targeted ways in large cohort studies (e.g. for QC of SVs in), though less commonly for all SVs in this setting; and (iv) in other situations. The authors seem to particularly emphasise (i). In some situations, particularly cancer cohorts, it is highly impractical to manually validate all predictions. In this situation, a tool for easily inspecting key predictions (potential key drivers, suspected false positive variants, or a sample for QC) is useful.

The samplot software has a well-designed interface, and I was able to easily install and run it. The plots generated are clear and it is apparent that they are a useful format. It should be noted that similar plots have been used previously, but are typically generated by unpublished code. There is no standard package supporting this activity. Samplot calculates read depth and identifies SV-supporting reads. A shortcoming of the manuscript that should be addressed is that the criteria for selection as a discordant read is not explained.

Example samplots are given in Figure 1 (multiple technologies revealing a DEL), and Figure 2 (examples of a duplication, inversion and translocation). These show the main SV types, but explanation of what is contained in these images is limited. From the caption of Figure 2, color is used to encode read pair orientation but this is not adequately explained in the manuscript. Figure 2C also requires more explanation. It appears there is one read supporting this SV. Is this a true positive? Examples of false positives, would be useful.

The idea of samplot-ML is interesting and to my knowledge quite novel, but also its development is limited. It is currently applicable to deletions only, which severely limits its utility. I'd consider that it would be useful in germline studies, where deletions are frequent, but would have concerns about relying on it in somatic studies, where there are complex and true deletion-like SVs from intrachromosomal translocations that may lack copy number changes. The simulated somatic testing is overly simplistic and does not capture realistic mutations or diverse cancer mutation types. Until substantially more testing is done across large numbers of cancers with different types of mutation, it would be better to avoid or moderate claims that it is useful for somatic samples.

The manuscript would benefit from more detail on training the model. The authors claim the model is robust to running on new datasets, but is retraining something a user would ever need to consider? The preprocessing of training and test data with image-magick seems fraught.

The authors state that samples in the test set may have contributed some information into the training set. Please clarify the nature of this "contamination". Is this via distant relationships, or some other mechanism? This may be unavoidable, but more information is needed to assess this statement. Can the relationships between these samples, or proportion of SVs shared be estimated?

The authors have taken care to generate a useful negative training set. My only concern with this is that complex SVs in cancer can resemble messy regions in the reference genome. This could negatively impact application in real cancer samples.

The manuscript is generally well written. I found two claims in the manuscript over-stated:

p1, Abstract: "Visual validation is an essential step to minimize false positive predictions…" This is study dependent. It may be essential in some studies, but is impossible in other studies (e.g. large cancer cohorts). I'd suggest important rather than essential.

p2, Background: "…any imprecision in the SV breakpoints will affect the visualisation making it impossible to differentiate between and absent SV and a slightly incorrect call." I would agree a slightly incorrect call makes assessing the simulated rearrangements more difficult to assess, and sometimes impossible, but in my experience, this is not always the case. This should be described in a more nuanced and accurate manner.

SV prediction is a highly challenging problem, and the community needs to continue developing, exploring and publishing new approaches to improve calling, qc, and data exploration and visualisation. Samplot is a simple but in my view useful and well-crafted tool for examining the evidence of an SV. It will prove useful in multiple ways. Samplot-ML represents an interesting and somewhat promising approach to reducing the number of false positives. It remains in the early stages of development. I am sceptical but open minded about its broader utility, especially in cancer.

In my view, samples is deserving of publication after addressing the above comments. I'd encourage a slightly more diverse view of how samples might be applied. I would recommend samplot-ml be framed as a tool for germline use only at this stage, and that somatic application be cautioned against or described as preliminary

**Authors Response**

**Point-by-point responses to the reviewers’ comments:**

Reviewer #1: Review of Belyeu. et al.

The authors present a method, SamPlot, to render and validate SVs either manually or with machine learning. I have been using SamPlot in my research and confirm the manual curation is easy to install and use, and is useful. Normally we would use IGV in a batch mode, but it is painfully slow, and the renderings of samplot are easier to view. Most of my comments are minor, with the one major comment regarding the comparison with duphold.

*We are excited that samplot is already part of your research, and thank you for your helpful comments.*

Comment 1. (minor) After stating SamPlot-ML only supports deletions, it is stated that other SV classes will be supported in the future. Because this is speculation, it should be moved to the discussion. You can use the active form to introduce SamPlot-ML: We developed SamPlot-ML to classify deletion SV calls …

Other statements in the manuscript are forward looking, and should not be used unless in the discussion, for example, "We are exploring other training strategies, including using regions that appear to be mutated from short-read data but are normal in long-read data." in the methods.

*You are right. We moved the text about support for other SV types and other future directions to the discussion.*

Comment 2 (minor) The figure 4A would be more interpretable as confusion matrices -- what are the SamPlot-ML classifications relative to GIAB?

*In addition to the raw stats provided in Supplementary Table 1, we have now provided confusion matrices (added as Supplementary Table 6) for all samples, callers, and filtering methods. The original stats in Supplementary Table 1, reported True Positives, False Positives, and False Negatives. False Negatives in this context refer to the number of SVs present in the truth set but absent from the callset. For our confusion matrices, we focus on the filtering methods’ capabilities, so we will define a False Negative as a true SV in the original callset that was wrongly filtered out. A True Negative will refer to a false positive SV in the original callset that was correctly filtered out.*

Comment 3 (minor) Typo - CHM1/CHM3 are stated to be "diploid". You'll want to say they are homozygous diploid, or 46,XX. Most of the time it is referred to as a haploid cell line which is technically incorrect.

*Good catch. We have corrected this inaccuracy.*

Comment 4 (minor) Conclusions should be folded into discussion.

*Agreed. The section titled “Conclusions” has been added to the end of the Discussion.*

Comment 5 (major). While the performance of paragraph and SV2 are surprisingly abysmal and sufficiently presented, the comparison with duphold is somewhat broad. For example, if parameters of duphold could be changed to reduce true positives from 1.1% to 2.4%, how many false positives are removed? Are there any particular regions where the ML approach correctly classifies a call when duphold does not? Additional effort should be made to highlight these regions (overlapping mobile elements/ tandem repeats / segmental duplications , genes, etc.).

*We were also surprised about SV2’s performance. In the preparation of our manuscript, we reached out to the SV2 authors for help determining the issue. No solution was found and the senior author of SV2 suggested we proceed with our results as is. Paragraph was less surprising given its (and all graph aligners) sensitivity to breakpoint resolution.*

*The percent reduction in False Positives for duphold when its True Positive reduction is 2.4% is approximately 35.93%.*

*To further address this point, we created ROC curves for each of the experiments involving duphold/DHFFC (pasted below and added as Supplemental Figure 10. We also created plots showing reduction in False Positives vs reduction in True Positives across all samples and SV callers presented (pasted below and added as Supplemental Figure 11. While duphold was tuned on HG002 (in blue), the 0.7 threshold is robust and nearly optimal in all of the samples we tested.*

*Regarding any patterns in the SVs that are missed by duphold, we could not find any that differed from the overall distribution of the larger SVs callset. This included size and overlap with repeat elements (e.g., self chains, segmental duplications, simple repeats, and other common repetitive elements). From visual inspection, it appears that the natural variation in coverage confused duphold’s DHFFC metric.*

Comment 6 (minor/major). The long read data is presented as "proof of concept". To me, this does not mean ready for publication, and due to the importance of having a tool to validate/curate calls from large sequencing projects such as TOPMed, the paper stands on it's own with short read data. It is up to the editors to decide if proof of concept results are appropriate to publish.

*We agree that “proof of concept” results can be below the publishable threshold. In the case of our long-read model, the performance, while not as good as the short-read model, is a valuable contribution to the field as projects like TOPMed that are starting to explore long-read SVs. We have therefore removed the “proof of concept” description, left the explanation for the reduced performance, and moved the discussion of our expectation of improved performance to end.*

Reviewer #2: The authors describe their software, samplot, for visualising alignment-and read depth-based evidence of structural variants. Samplot supports manual visual curation of SV calls, which can be employed to reduce false positives in some situations. Motivated by the idea of visually inspecting supporting evidence, the authors have also developed a machine learning model, samplot-ml, to automate the removal of false positive calls. This uses a convolutional neural network to perform classification on the images produced by samplot. Samplot-ml currently supports deletions only. A proof-of-concept model was also developed for long reads.

*We thank the reviewer for their insightful comments, and have numbered the comments to make it easy to reference in our revision.*

1. In my view, samplot is a useful tool for when the evidence for an SV needs to be inspected.

This is a task that arises (i) in the wide-scale manual curation of SVs from an individual sample—an approach that is sometimes applied; (ii) in targeted ways to look at potential causal variants in trio studies; (iii) in targeted ways in large cohort studies (e.g. for QC of SVs in), though less commonly for all SVs in this setting; and (iv) in other situations. The authors seem to particularly emphasise (i). In some situations, particularly cancer cohorts, it is highly impractical to manually validate all predictions. In this situation, a tool for easily inspecting key predictions (potential key drivers, suspected false positive variants, or a sample for QC) is useful.

*We are excited that the reviewer thinks that samplot is useful. While we agree that these are the settings where samplot is used, we disagree that it is impractical to manual validate large cohorts. Samplot was designed for this use case and we and others have used it to curate thousands (Belyeu at el, 2021, in press at AJHG) to hundreds of thousands of SVs from a single study (Bertolotti, 2020, Nature Communications). Several ongoing studies with hundreds of samples each are currently using samplot, and we have found it particularly useful in non-human genomes where the reference genome is less mature and false positives are more common. Also, see response to comment 9.*

2. The samplot software has a well-designed interface, and I was able to easily install and run it. The plots generated are clear and it is apparent that they are a useful format. It should be noted that similar plots have been used previously, but are typically generated by unpublished code. There is no standard package supporting this activity. Samplot calculates read depth and identifies SV-supporting reads. A shortcoming of the manuscript that should be addressed is that the criteria for selection as a discordant read is not explained.

*We are happy to hear that our work to make the software usable and reproducible is paying off.*

*The definition of a discordant pair is an essential one. We have added a new Supplemental Note 1 and Supplemental Figure 8 (also below) which describe in depth how these reads are defined and identified, cited in the fifth paragraph of the Results section. One important clarification that we included in Supplemental Note 1 is that samplot does not identify supporting reads. All of the discordant reads in a region are displayed and the user can decide if the SV has support.*

3. Example samplots are given in Figure 1 (multiple technologies revealing a DEL), and Figure 2 (examples of a duplication, inversion and translocation). These show the main SV types, but explanation of what is contained in these images is limited. From the caption of Figure 2, color is used to encode read pair orientation but this is not adequately explained in the manuscript. Figure 2C also requires more explanation. It appears there is one read supporting this SV. Is this a true positive? Examples of false positives, would be useful.

*As mentioned above, we have added a new Supplemental Note 1 and Supplemental Figure 8 (also above) which describe the selection of split and discordant reads in detail, as well as the color-coding used in samplot images. Figure 2C shows an example of a true positive inverted translocation. While separate reads are not distinguishable, the transparency value of read plots indicates when many vs few reads are shown together with the same coordinates. We have clarified this in the legend for Figure 2. New examples of false positive deletion, duplication, and inversion variant calls have been added in a new Supplemental Figure 5.*

4. The idea of samplot-ML is interesting and to my knowledge quite novel, but also its development is limited. It is currently applicable to deletions only, which severely limits its utility. I'd consider that it would be useful in germline studies, where deletions are frequent, but would have concerns about relying on it in somatic studies, where there are complex and true deletion-like SVs from intrachromosomal translocations that may lack copy number changes. The simulated somatic testing is overly simplistic and does not capture realistic mutations or diverse cancer mutation types. Until substantially more testing is done across large numbers of cancers with different types of mutation, it would be better to avoid or moderate claims that it is useful for somatic samples.

*We are happy to hear that the reviewer finds our work interesting and novel.*

*We agree with the reviewer that our current support for only deletions is a limitation since many other SV types occur and can drive disease traits. As discussed in the paper, this limitation is solely due to the limited number of non-deletion high-quality truth sets. Duplications, inversions, and translocations can all be visualized by Samplot and create distinct visual signatures (see Figures 1-2 and Supplemental Figures 6-7) that tend to yield good models. The current truth sets contain a small number of these events. While we could train a model on these data, in our opinion, it is better to wait. As the Human Genome SV Consortium and other projects expand their long-read programs to include more duplications and copy-neutral SVs in their high-quality truth sets, we too can expand our support.*

*As for the applicability of Samplot-ML to somatic SV detection, we agree with the reviewer that our support for only deletions will not help with complex copy-neutral events. As the reviewer accurately states, Samplot-ML would require specific training to correctly classify deletion-like chromosomal “cut-and-paste” translocations, as these translocations are not true deletions. If correctly called and annotated as translocations, this presents no issue for Samplot-ML, as the trained deletion model will not attempt to classify a translocation. If a translocation is improperly identified as a deletion, Samplot-ML will classify it as a false positive. This classification is accurate, as a miscalled translocation would be a false positive deletion call, but could result in de-prioritization of intrachromosomal translocations. A warning to this effect has also been added to the final paragraph of the Results section.*

*However, while other SV types occur more frequently in some cancers than in the germline, deletions remain one of the most common SV classes across cancer types (Li et al. 2020). Losses of tumor suppressors and gene fusions (e.g., EGFR), both often created by deletions, are a significant focus of cancer diagnostics and drive treatment decisions. The improvements that Samplot-ML provides over other methods for curating deletion calls can be extremely useful in tumor studies, as they are in germline studies, albeit in some cases, to a lesser degree. Despite the importance of deletions and the improvements that Samplot-ML provides, we agree that somatic/tumor-derived variants are a distinct and difficult problem, especially in cases where SVs are comprised of multiple or complex rearrangements, and readers should be aware of the issue. We have added an additional caveat expressing this potential shortcoming in the final Results paragraph.*

*In addition to complex SVs, a major challenge in somatic SV detection is tumor clonality and its effect on an SV's allele balance. Many bioinformatics tools assume the target sample is diploid (i.e., two copies of each chromosome) and that the proportion of reads matching the alternate allele will be roughly 0, 0.5, or 1 (depending on the number of affected chromosomes), which is often not the case in heterogeneous tumors. When SVs arise in clones, the proportion of reads in bulk sequencing that match the alternate allele will depend on that clone's frequency. For example, a heterozygous SV in a clone at 25% frequency will appear to have an allele balance of 0.125. Genotyping software that assumes a diploid genome is likely to classify this SV as a false positive. Our experiment, which we agree is simple and takes a reductionist approach, is intended only to measure the effect that variable alternate read frequency has on our model since it too was trained on diploid samples. This experiment was in direct response to feedback we received from cancer researchers, and we believe it offers insight into the detection limits of Samplot-ML and other tools.*

5. The manuscript would benefit from more detail on training the model. The authors claim the model is robust to running on new datasets, but is retraining something a user would ever need to consider? The preprocessing of training and test data with image-magick seems fraught.

*Our goal is to have two general-purpose models, one for short reads and one for long reads, that perform well on nearly every data set (see described in the second paragraph of the Results subsection titled “Automated SV curation with Samplot-ML”). In our experience, the adoption of any tool drops precipitously when users are required to generate their own resources, and as we discuss in the paper, curating a training set is not easy. That said, there will undoubtedly be experimental designs where our models are not appropriate. For these instances, we have a completely documented and reproducible training process that others can use as a template for their specific needs. For example, long-read sequencing and classifying different SV types, will require retraining and will likely constitute much of the future development of Samplot-ML.*

*The process of image preprocessing before training is a necessary step to avoid potential overtraining on uninformative plot aspects such as text and axes and is a standard step in image-based learning models. The preprocessing step is also applied to any test data to ensure the same training and testing conditions. We agree that this step could be difficult to reproduce, which is why we wrapped the cropping in a dedicated cropping script that is part of our open-source repository.*

6. The authors state that samples in the test set may have contributed some information into the training set. Please clarify the nature of this "contamination". Is this via distant relationships, or some other mechanism? This may be unavoidable, but more information is needed to assess this statement. Can the relationships between these samples, or proportion of SVs shared be estimated?

*The reviewer is right, we need to expand on this contamination. While we removed direct familial relationships from the training set, other distantly related individuals remain. Given their shared ancestral histories, some SVs in the test set also appeared in the training set. Out of 8674 DEL SVs across HG00514, HG00733, NA19240 callsets, 78 (< 1%) appeared in the 1000 genomes callset after excluding direct relatives.*

7. The authors have taken care to generate a useful negative training set. My only concern with this is that complex SVs in cancer can resemble messy regions in the reference genome. This could negatively impact application in real cancer samples.

*This is a valid concern. We have added a caveat to the last paragraph of the Results section to inform readers of potential issues in somatic SV curation.*

8. The manuscript is generally well written. I found two claims in the manuscript over-stated:

9. p1, Abstract: "Visual validation is an essential step to minimize false positive predictions…" This is study dependent. It may be essential in some studies, but is impossible in other studies (e.g. large cancer cohorts). I'd suggest important rather than essential.

*We respectfully disagree with the reviewer on this point. In all of our SV analysis work, including clinical (Brady et al. 2017; Werling et al. 2018), human population (Belyeu et al. 2020), and non-human population (Bertolotti et al. 2020), we relied heavily on manual inspection, especially for SVs that we suspect have phenotypic consequences. Samplot was born out of this need, and its primary design goal was to scale to large studies. For example, we used samplot to manual inspect over 100,000 SVs among over 400 salmon samples. Several on-going projects that include hundreds of samples are also using samplot.*

10. p2, Background: "…any imprecision in the SV breakpoints will affect the visualisation making it impossible to differentiate between and absent SV and a slightly incorrect call." I would agree a slightly incorrect call makes assessing the simulated rearrangements more difficult to assess, and sometimes impossible, but in my experience, this is not always the case. This should be described in a more nuanced and accurate manner.

*This is a good point and we have corrected that statement.*

11. SV prediction is a highly challenging problem, and the community needs to continue developing, exploring and publishing new approaches to improve calling, qc, and data exploration and visualisation. Samplot is a simple but in my view useful and well-crafted tool for examining the evidence of an SV. It will prove useful in multiple ways. Samplot-ML represents an interesting and somewhat promising approach to reducing the number of false positives. It remains in the early stages of development. I am sceptical but open minded about its broader utility, especially in cancer.

*It is encouraging and motivating that the reviewer finds samplot to be a high-quality tool.*

12. In my view, samples is deserving of publication after addressing the above comments. I'd encourage a slightly more diverse view of how samples might be applied. I would recommend samplot-ml be framed as a tool for germline use only at this stage, and that somatic application be cautioned against or described as preliminary

*We have added a statement clarifying that Samplot-ML is primarily intended for use in germline SV callsets.*

*References*

*Belyeu, J. R., H. Brand, H. Wang, X. Zhao, and B. S. Pedersen. 2020. “De Novo Structural Mutation Rates and Gamete-of-Origin Biases Revealed through Genome Sequencing of 2,396 Families.” bioRxiv. https://www.biorxiv.org/content/10.1101/2020.10.06.329011v1.full-text.*

*Bertolotti, Alicia C., Ryan M. Layer, Manu Kumar Gundappa, Michael D. Gallagher, Ege Pehlivanoglu, Torfinn Nome, Diego Robledo, et al. 2020. “The Structural Variation Landscape in 492 Atlantic Salmon Genomes.” https://doi.org/10.1101/2020.05.16.099614.*

*Brady, Samuel W., Jasmine A. McQuerry, Yi Qiao, Stephen R. Piccolo, Gajendra Shrestha, David F. Jenkins, Ryan M. Layer, et al. 2017. “Combating Subclonal Evolution of Resistant Cancer Phenotypes.” Nature Communications 8 (1): 1231.*

*Werling, Donna M., Harrison Brand, Joon-Yong An, Matthew R. Stone, Lingxue Zhu, Joseph T. Glessner, Ryan L. Collins, et al. 2018. “An Analytical Framework for Whole-Genome Sequence Association Studies and Its Implications for Autism Spectrum Disorder.” Nature Genetics 50 (5): 727–36.*

**Second round of review**

**Reviewer 1**

My comments were sufficiently addressed

**Reviewer 2**

The authors have done a thorough job of addressing my concerns. I would support the publication of the manuscript.
